# A deep learning algorithm to identify cervical ossification of posterior longitudinal ligaments on radiography

**DOI:** 10.1038/s41598-022-06140-8

**Published:** 2022-02-08

**Authors:** Koji Tamai, Hidetomi Terai, Masatoshi Hoshino, Akito Yabu, Hitoshi Tabuchi, Ryo Sasaki, Hiroaki Nakamura

**Affiliations:** 1 Department of Orthopedics, Osaka City University Graduate School of Medicine, 1-5-7, Asahimachi, Abenoku, Osaka city, Osaka 545-8585 Japan; 2 Department of Technology and Design Thinking for Medicine, Hiroshima University, Hiroshima, Japan; 3 Department of Ophthalmology, Tsukazaki Hospital, Himeji, Japan

**Keywords:** Neurology, Signs and symptoms

## Abstract

The cervical ossification of the posterior longitudinal ligament (cOPLL) is sometimes misdiagnosed or overlooked on radiography. Thus, this study aimed to validate the diagnostic yield of our deep learning algorithm which diagnose the presence/absence of cOPLL on cervical radiography and highlighted areas of ossification in positive cases and compare its diagnostic accuracy with that of experienced spine physicians. Firstly, the radiographic data of 486 patients (243 patients with cOPLL and 243 age and sex matched controls) who received cervical radiography and a computer tomography were used to create the deep learning algorithm. The diagnostic accuracy of our algorithm was 0.88 (area under curve, 0.94). Secondly, the numbers of correct diagnoses were compared between the algorithm and consensus of four spine physicians using 50 independent samples. The algorithm had significantly more correct diagnoses than spine physicians (47/50 versus 39/50, respectively; *p* = 0.041). In conclusion, the accuracy of our deep learning algorithm for cOPLL diagnosis was significantly higher than that of experienced spine physicians. We believe our algorithm, which uses different diagnostic criteria than humans, can significantly improve the diagnostic accuracy of cOPLL when radiography is used.

## Introduction

Cervical ossification of posterior longitudinal ligament (OPLL) can result in spinal canal or foraminal narrowing, cause by myelopathy or radiculopathy, and increase spinal cord injury risk following a traumatic event^1^. It is a multifactorial, degenerative disease, and both environmental and genetic factors contribute to its development, type, and severity^2^. The prevalence of cervical OPLL detected by the cervical radiography has been estimated as 2% in Japan, 0.12% in the United States, and 0.10% in Germany^3^. In contrast, the prevalence of cervical OPLL detected by computed tomography (CT) has been estimated as 6.3% in Japan and 2.2% in the United States^4,5^.

Since OPLL is a progressive disease^6^, an accurate diagnosis in the early phase is crucial. However, one of the intractable problems associated with OPLL treatment is its misdiagnosis/overlook on radiography^7^. The abovementioned differences between OPLL prevalence when detected on radiography and CT scans directly illustrate this problem. Recently, a standard method for OPLL diagnosis that involves CT scanning rather than radiography has been suggested^7^. However, medical radiation exposure due to the CT scan is a drawback of the method^8,9^. Accordingly, physicians should avoid routine CT scans in patients with cervical symptoms.

A deep learning algorithm to detect cervical OPLL on cervical radiography has the potential to assist physicians by decreasing misdiagnosis rates and facilitating the implementation of timely therapy in patients with early-phase OPLL. Furthermore, the use of the algorithm will improve patient safety by minimizing radiation exposure, as cervical radiography has been determined to be associated with 1/700 times the radiation exposure of a CT scan^10^.

Therefore, this study aimed to validate the diagnostic yield of our deep learning algorithm for detecting cervical OPLL on radiography and compare its diagnostic accuracy with that of experienced spine physicians.

## Results

### Demographics

No significant differences were noted in the average age and number of females/males between OPLL patients and controls (*p* = 0.891 and 1.000, respectively; Table 1). Among all patients, 224 were from institution A, 166 were from institution B, and 96 were from institution C. Regarding the type of OPLL, the mixed type was most prevalent (n = 110, 45.3%), followed by the segmental type (n = 67, 27.6%), localized type (n = 44, 18.1%), and continuous type (n = 22, 9.1%). The OPLLs were mostly located in the middle-to-lower cervical levels (n = 86, 35.4%), followed by the middle cervical (n = 67, 27.6%), upper-to-middle (n = 65, 18.1%), and whole cervical levels (n = 25, 10.3%).

**Table 1:** Demographic data.

	OPLL group	Control group	*p*-value
Number of patients	243	243	
Average age	63.5 ± 10.1	64.9 ± 11.2	0.891#
**Sex**			0.850*
Female	86	89	
Male	157	154	
**Collected institution**			1.000*
Institution A	112	112	
Institution B	83	83	
Institution C	48	48	
**OPLL type**			
Continuous	22	–	
Segmental	67	–	
Mixed	110	–	
Localized	44	–	
**OPLL location**			
Upper-to-middle	65	–	
Middle	67	–	
Middle-to-lower	86	–	
Whole cervical	25	–	

#Mann–Whitney U test, *Chi-squared test.

OPLL, ossification of the posterior longitudinal ligament.

### Accuracy of the deep learning algorithm

**Figure 1:**
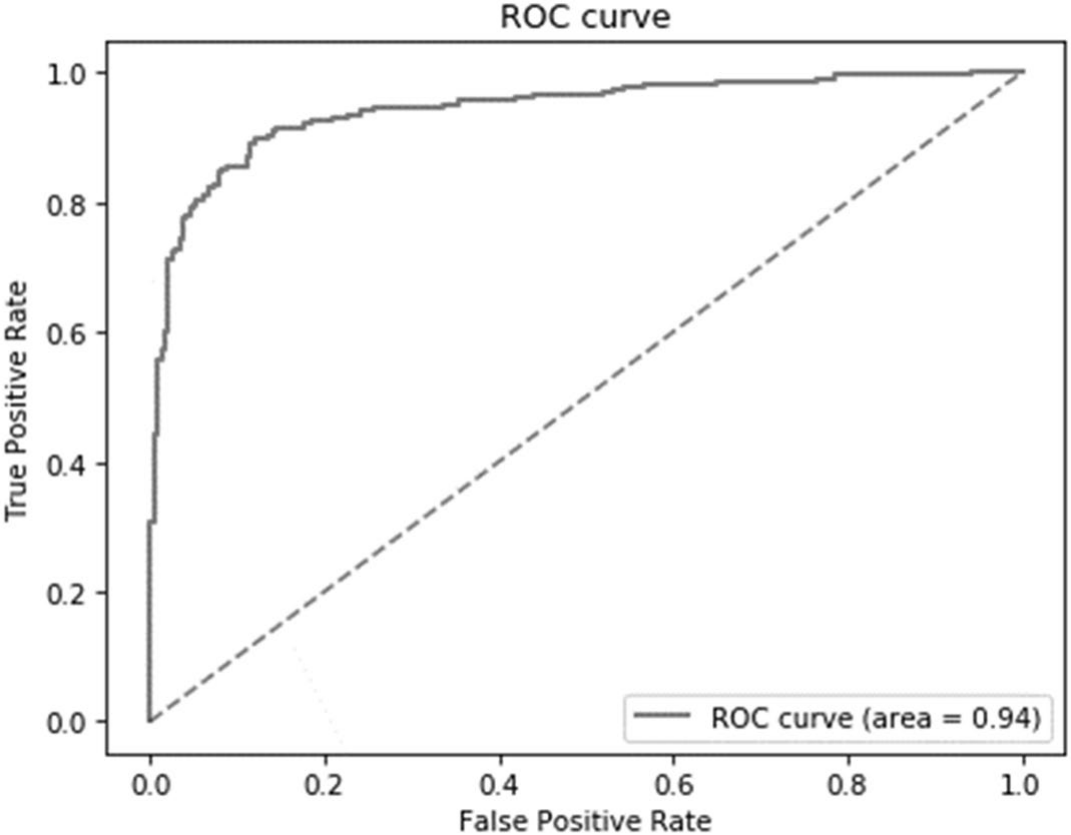
The ROC curve of the diagnostic accuracy of the deep learning algorithm is shown. ROC, receiver operating characteristic.

**Figure 2:**
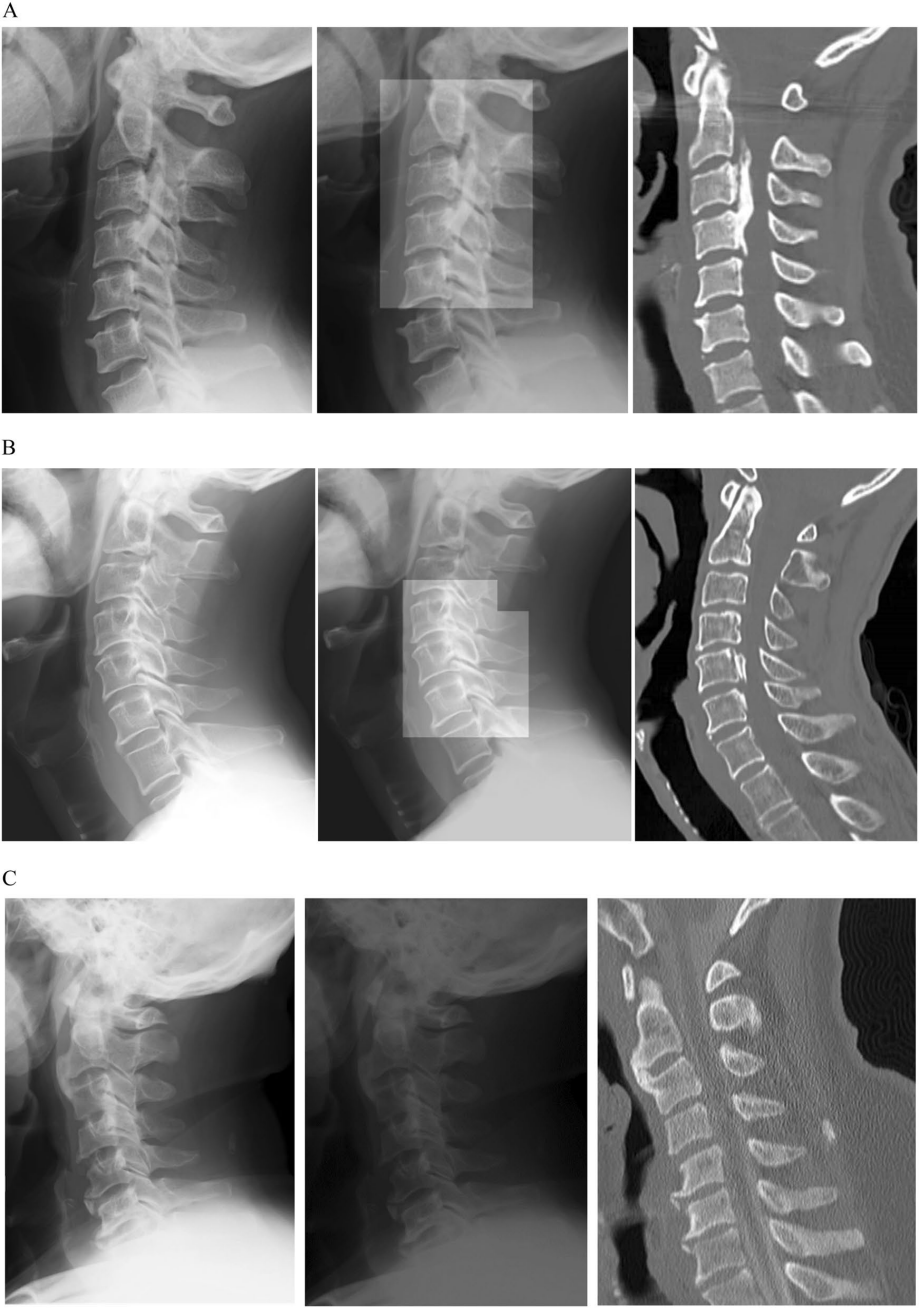
Representative images used and created by the deep learning algorithm are shown. The left image shows the cervical plain radiograph used in the deep learning algorithm. Images created by our algorithm are shown on the center. The right image shows a sagittal slice of the computed tomography image used as the ground truth, but not used in the algorithm. The algorithm was designed to highlight areas of suspected ossification of the posterior longitudinal ligament (OPLL) when OPLL was identified in an image. (**A**) An image from a 47-year-old women with a continuous-type OPLL from C2–C4 is shown. (**B**) An image from a 56-year-old man with a small segmental OPLL at C5 and C6 is shown. (**C**) An image from a 63-year-old man without cervical OPLL is shown.

The overall diagnostic accuracy, precision, and recall of our deep learning algorithm were 0.88, 0.86, and 0.90, respectively (Table 2). In the ROC analysis, the area under curve (AUC) of the presence/absence of OPLL was 0.94 (95% confidential intervals, 0.92–0.97; *p* < 0.001; Fig. 1). Representative images created by our algorithm are shown in Fig. 2. In the subgroup analysis based on the institution, accuracy was highest when images from institution B were considered and lowest when those of institution A were considered (0.95 versus 0.87, respectively; Table 2). In the subgroup analysis based on the OPLL type, recall was the highest for mixed-type OPLL and lowest for localized-type OPLL (0.96 versus 0.82, respectively). In the subgroup analysis based on the OPLL location, recall was highest at whole cervical levels, and lowest at the middle cervical level (1.00 versus 0.87, respectively).

**Table 2:** Diagnostic results of the deep learning algorithm (n = 486).

	TP	FP	FN	TN	Accuracy	Precision	Recall
Overall	219	34	24	209	0.88	0.86	0.90
**Institution**							
Institution A	99	22	15	92	0.85	0.82	0.87
Institution B	79	7	4	76	0.93	0.92	0.95
Institution C	41	5	5	41	0.90	0.89	0.89
**OPLL type**							
Continuous	20	–	2	–	–	–	0.91
Segmental	57	–	10	–	–	–	0.85
Mixed	106	–	4	–	–	–	0.96
Localized	36	–	8	–	–	–	0.82
**OPLL location**							
Upper to middle	60	–	5	–	–	–	0.92
Middle	58	–	9	–	–	–	0.87
Middle to lower	76	–	10	–	–	–	0.88
Whole cervical	25	–	0	–	–	–	1.00

TP, true positive; FP, false positive; FN, false negative; TN, true negative; OPLL, ossification of the posterior longitudinal ligament.

### Comparisons in the accuracy of the deep learning algorithm and spine surgeons

**Figure 3:**
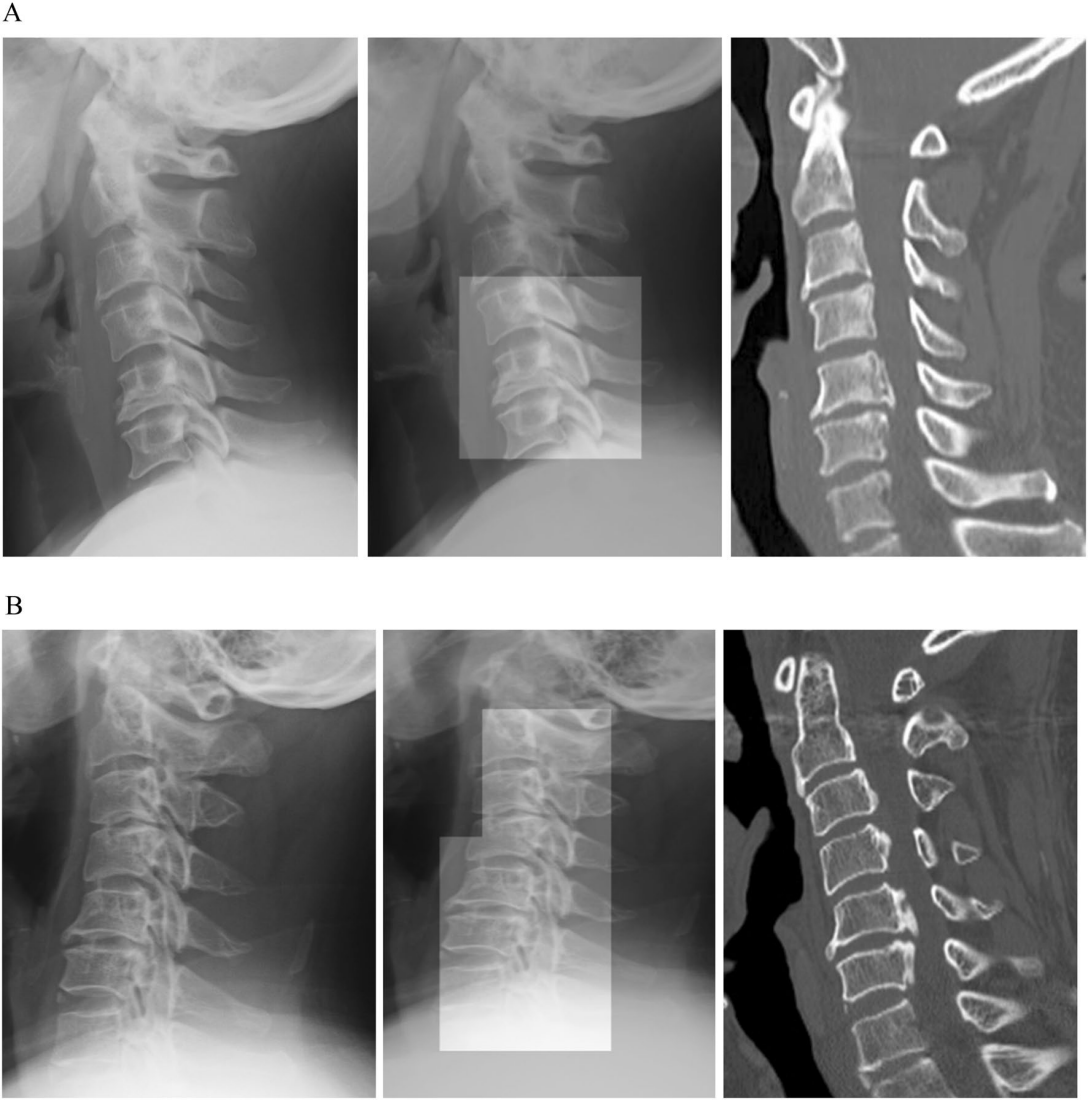
Images in which only the algorithm could identify an ossification of the posterior longitudinal ligament (OPLL) are shown. (**A**) An image from a 56-year-old woman with a small segmental OPLL at C5 is shown. (**B**) An image from a 72-year-old man with an OPLL at C5–C6 is shown.

The accuracy of our deep learning algorithms was 0.92, whereas that of the four spine surgeons was 0.80, 0.78, 0.76, and 0.74 (Table 3). Figure 3 depicts patients for whom all four surgeons failed to identify the OPLL, while the deep learning algorithm could accurately identify the OPLL. The number of correct assessments by the learning algorithm was significantly higher than that by the four surgeons (47/50 versus 39/50, respectively; *p* = 0.041, Table 4).

**Table 3:** Diagnostic accuracy of the deep learning algorithm and four spine surgeons (n = 50).

	TP	FP	FN	TN	Accuracy
Deep learning algorithm	24	1	2	23	0.92
Surgeon 1 (> 25 y exp.)	22	3	7	18	0.80
Surgeon 2 (> 20 y exp.)	20	5	8	17	0.74
Surgeon 3 (> 10 y exp.)	21	4	8	17	0.76
Surgeon 4 (> 5 y exp.)	23	2	9	16	0.78

TP, true positive; FP, false positive; FN, false negative; TN, true negative; y, years; exp, experience.

**Table 4:** Comparison of the diagnostic accuracy between the deep learning algorithm and the consensus of four spine physicians (n = 50).

	TP + TN	FP + FN	*p*-value	Accuracy
Deep learning algorithm	47	3	0.041*	0.92
Surgeons’ consensus	39	11		0.78

*Chi-square test.

TP, true positive; FP, false positive; FN, false negative; TN, true negative.

## Discussion

Overall, the diagnostic accuracy of the deep learning algorithm was 0.88, and the AUC was 0.94. However, the accuracy was affected by the following factors: institution at which radiographic images were obtained, OPLL type, and the segment-level of the OPLL. The deep learning algorithm performed significantly better than the consensus of experienced Japanese spine surgeons.

A strength of this study was its clear establishment of the ground truth by the presence or absence of cervical OPLL on CT. To create the deep learning algorithm, determination of the ground truth is a critical issue. For example, Won et al. created a convolution neural network (CNN) to classify lumbar canal stenosis severity into four grades^11^. Although their study was informative, the study methodology and results were complex; two radiologists assessed lumbar canal stenosis on magnetic resonance imaging independently, and two types of CNNs were investigated using the radiological findings determined by the radiologists. The agreement between the CNNs and radiologists were comparable to that between the two radiologists. A difficulty in the interpretation of this result arises from the ambiguousness of the ground truth of the previous study; namely, the stenotic grade (i.e., ground truth) was subjectively evaluated and differed by observer. In contrast, Maki et al. reported on a CNN that distinguished between spinal schwannoma and meningioma, with an accuracy value comparable to that of a professional radiologist^12^. Their study provided a clear message because the ground truth was a histological result assessed post-resection, comprising objective and consistent data. In the present study, we used cervical OPLL on CT as the ground truth, similarly comprising objective and consistent data.

In the current study, results of our algorithm may be affected by both OPLL type and the institution at which the cervical radiographic images were obtained. The potential reasons for the differences according to institution included the concentration of radiography, incidence angle of the X-ray, and patient positioning. Further improvement in the algorithm is warranted to provide consistent results regardless of the institution at which cervical radiography is performed.

Artificial intelligence cannot overcome human abilities^13^, as labeled training data and the ground truth for creating the algorithm must be set by a human. However, our algorithm could produce a significantly higher number of correct assessments regarding the presence/absence of OPLL on radiography than experienced Japanese surgeons, who routinely diagnose OPLL^2^. This performance improvement is due to the fact that the deep learning system was trained using not only cervical radiography but also CT data as reference. This procedure could be considered as a type of radiomics, which refers to a method used to extract a large number of features from radiographic images using data-characterization algorithms^14^. Radiomics significantly aids physicians to improve the efficiency and accuracy of their diagnoses and has even been used to predict prognoses by measuring and analyzing features of medical images. We do not think that our algorithm will be capable of automatically selecting patients with OPLL; however, the algorithm will suggest to physicians whether the presence/absence of OPLL is likely, while applying different diagnostic criteria from those used by physicians. For example, the recall of our algorithm to identify the OPLL located below C6 level was almost similar to the other level, although the human tended to miss the OPLL located in the lower cervical level due to overlapping of the shoulder line. The postulated reason of high recall of the algorithm may be segmentation. Namely, AI algorithm would evaluate the OPLL with extremely small segment rather than global perspective like humans do^15^. Hence, for human, the lower cervical level may be difficult to observe in comparison with the upper and middle cervical level; meanwhile, for AI algorithm, the shoulder line might not disturb to evaluate the OPLL in the lower cervical level. With this example in mind, the use of the AL algorithm which have different diagnostic criteria could potentially improve the physician’s diagnostic yield of OPLL.

This study has the potential to impact physicians and patients in the clinical setting. Importantly, the misdiagnosis/overlook of OPLL is expected to decrease with the use of our deep learning algorithm. This is because the algorithm both suggests the presence/absence of OPLL and highlights suspected lesions on radiography. This benefits patients by providing adequate examinations or therapies throughout the relatively early phases of OPLL. Additionally, the use of the algorithm would increase patient safety by minimizing radiation exposure, as the algorithm can effectively identify OPLL using only cervical radiography. Finally, our algorithm could contribute to not only spine physicians but also primary doctors, emergency doctors, and orthopedic physicians who may have chances to take cervical radiography in their daily clinical setting.

Our deep learning algorithm had several limitations. First, to distinguish OPLL with some type of osteophyte is difficult. The OPLL in the current study was defined as “the ossification of the posterior longitudinal ligament with more than 2 mm thickness in the axial CT image” based on the previous report^4^. However, we may miss the OPLL which is less than 2 mm and/or may include the large osteophyte which occurred from the posterior corner of vertebra. Second, all cervical radiographic images were collected from the Japanese population. Although no major differences between the Japanese and other races have been observed, several minor differences, such as the spinal canal diameter, may be crucial parameters considered within the deep learning algorithm^16,17^. Third, postoperative images were excluded when the algorithm was established. Since it is well-known that the some OPLLs would progress after surgery, an algorithm that can detect OPLL with postoperative radiographic images may be of use to physicians^18^. Fourth, as mentioned previously, the results of our algorithm were affected by the OPLL type and location and the institution at which the cervical radiography was performed. Fifth, though we used a k-fold cross-validation technique, which allows for an efficient validation of small datasets without requiring separate test data^19^, a larger sample size would be ideal for creating a more precise algorithm. Finally, the current cross-sectional study design cannot determine the risk of future OPLL growth. To overcome these limitations, an international, longitudinal, large-scale study with precise clinical scores is warranted.

## Conclusion

We created a deep learning algorithm capable of suggesting the presence/absence of OPLL on cervical radiography and highlighting suspected areas of ossification on radiographic images when an OPLL is identified. The diagnostic yield of the algorithm for cervical OPLL on radiography was higher than that for the consensus of experienced spine physicians. We believe our algorithm, which uses different diagnostic criteria than humans, can significantly improve the diagnostic accuracy of OPLL when radiography is used.

## Methods

### Study design and ethics

We performed a cross-sectional study of patients who received cervical radiography and a CT scan. All study participants provided written informed consent. The study was performed in accordance with the World Medical Association Declaration of Helsinki^20^.

### Collection of data

Data were collected from a database that included patient records from three institutions. Inclusion criteria were as follows: patients who underwent radiography for symptoms such as neck pain, radiculopathy, neurological deficits, or cervical deformity and patients who received cervical high-resolution CT and plain radiography within a 3-month interval. Exclusion criteria were as follows: patients who underwent previous cervical surgery; patients who did not consent to the use of their data for study purposes; and patients with obvious spinal tumors or trauma. Patients with OPLL were identified using CT images regardless of myelopathic symptom occurrence. Finally, 243 patients were included in the OPLL group. To identify matched controls from the database, 1:1 propensity score matching was performed. We fit a logistic regression model using patient age, sex, and institution to estimate a propensity score, and a nearest-neighbor matching procedure was performed. After matching, the data of 243 patients were extracted for use as control. The absence of cervical OPLL using CT images was confirmed in control patients.

### Labeling process

**Figure 4:**
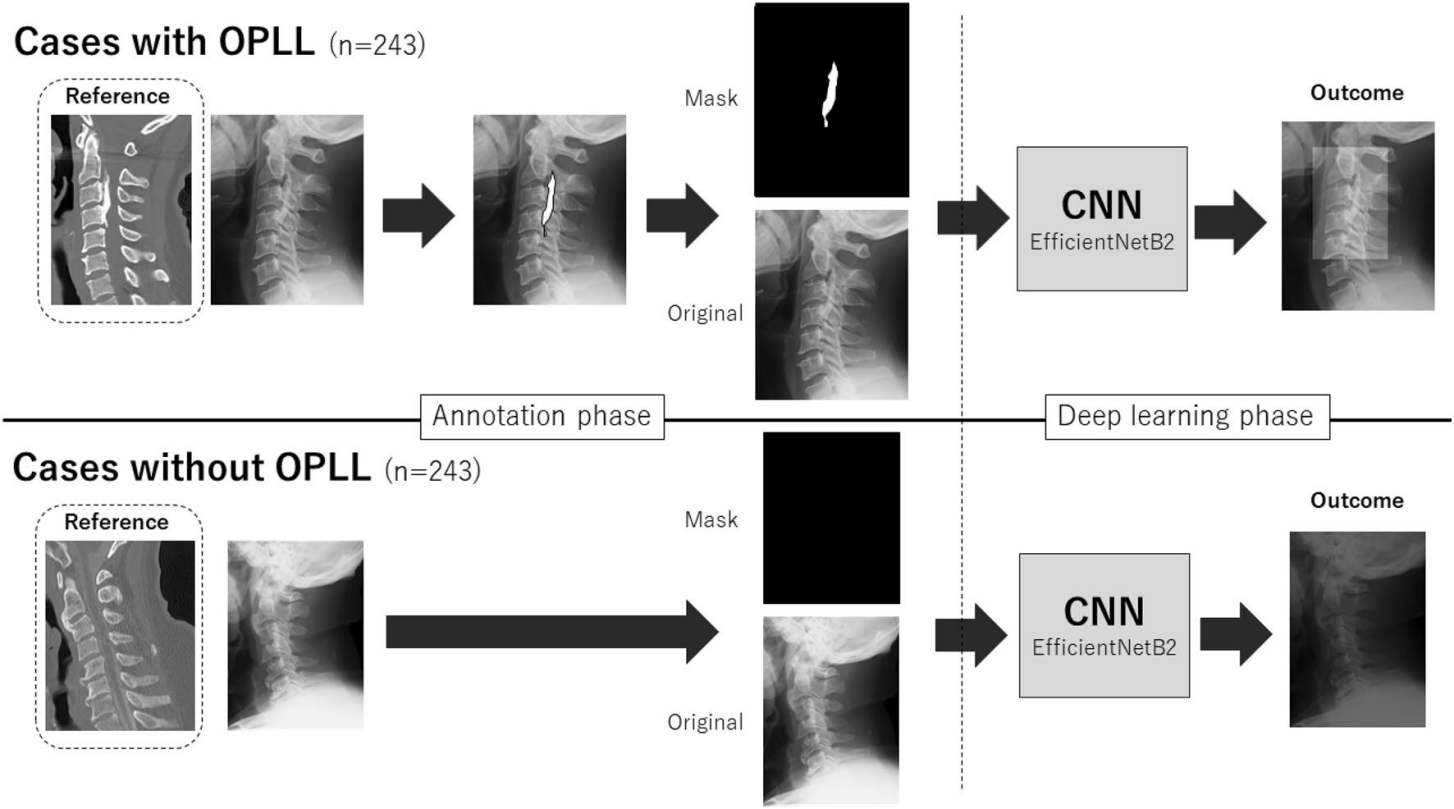
Illustration of study process. Lateral cervical plain radiographies of all patients were extracted as jpeg files from the DICOM database. As annotation phase, an independent researcher manually painted the ossification area in the cases with OPLL on jpeg images of radiography with the reference of CT images. Subsequently, the painted image was divided into mask images for ground truth and original image, and both were used to construct the CNN. In the cases without OPLL on referenced CT image, all-black mask images were created as ground truth for CNN.

Lateral cervical plain radiographies of all patients were extracted as 224 × 224-pixel jpeg files from the DICOM database after personal information was removed. Independent spine surgeon manually painted the ossification area on jpeg images using computer software (e-Growth Co., Ltd.; Kyoto, Japan). Based on the previous definition, the OPLL was defined as ossification of the posterior longitudinal ligament with more than 2 mm thickness in the axial plane CT image^4^. During this procedure, the spine surgeon used sagittal, axial, and three-dimensional reconstructed CT images as reference to identify the precise shape and extent of ossification on cervical radiographic jpeg images (Fig. 4).

### Establishment of the deep learning algorithm

To increase the quantity of training data available, data augmentation techniques such as inversion, equalization, brightness, gamma correction, histogram, noise addition, and mix-up were applied to the images within the training dataset. Subsequently, a CNN model was constructed and trained using sub-images randomly cropped at 224 × 224 pixels from preprocessed image data. Using amplified images, we constructed a model to highlight suspected ossification using a CNN model called EfficientNetB2^21^. Ten-fold cross validation was performed to establish the algorithm. To accomplish this, all jpeg images were equally divided into 10 groups, and 9 of the 10 groups were used for training, whereas the remaining group was used for model validation. This process was repeated 10 times so that the groups were adequately assessed^19^. Model construction and validation were carried out using Keras (https://keras.io/en/), which runs Python’s TensorFlow backend (https://www.tensorflow.org/). Training and validation of the CNN were performed using a computer with a GeForce GTX 1080 Ti (NVIDIA, Santa Clara, CA) graphics processing unit.

### Algorithm validation

Using patient data from all 486 individuals, cases of true positive (TP), false positive (FP), false negative (FN), and true positive (TN) were counted. Then, the following parameters were calculated: accuracy, defined as “(TP + TN)/ (TP + FP + FN + TN)”; precision, defined as “TP/(TP + FP)”; and recall, defined as “TP/(TP + FN)”. Sub-analyses were performed according to the individual institution, OPLL type, and OPLL location. In the analyses based on the OPLL type or OPLL location, only the OPLL group was included, and only recall was calculated.

### OPLL classification

Cervical OPLL was classified into four types based on a classification system established by the Japanese Ministry of Health, Labor, and Welfare using CT images^22–24^: continuous, a long lesion extending over several vertebral bodies; segmental, one or several separate lesions behind vertebral bodies; mixed, a combination of continuous and segmental types; and circumscribed, mainly located posterior to the disc space. The location of cervical OPLL was defined as follows: upper-to-middle cervical level (OPLLs mainly found between the C2 and C4 levels); middle cervical level (OPLLs mainly found between the C5 and C6 levels); middle-to-lower level (OPLLs mainly found below the C6 level); and whole cervical levels (OPLLs found throughout whole cervical levels from C2 to below C6).

### Comparison with surgeon assessments

The deep learning algorithm and four spine surgeons (HT, HM, AY, and RS with > 25, > 20, > 10, and > 5 years of experience, respectively) independently evaluated 50 cervical radiographic jpeg images for the presence or absence of OPLL (25 patients with OPLL and 25 patients without OPLL). Surgeons were allowed to use software functions to expand the images and control the image tone. After surgeons independently evaluated the images, a consensus was reached. When three out of four surgeons agreed, the assessment reached by the majority was considered as the consensus assessment; when the evaluator’s assessments were split evenly (2:2), the surgeons discussed the findings until a consensus could be reached.

### Statistical analysis

The chi-square or Fisher’s exact test were used to compare categorical variables and the Mann–Whitney U test for continuous variables. To evaluate the diagnostic accuracy of our algorithm, the receiver operating characteristic (ROC) curve and AUC were calculated. All analyses were performed using SPSS version 23 software (IBM Corp., Armonk, NY, USA). *P*-values < 0.05 were considered statistically significant.

### Ethical approval and informed consent

IRB approval: All study participants provided informed consent, and the study protocol was approved by the Institutional Review Board of Osaka City University (No. 3170).
